# Supplementary material for: Development and characterization of new tools for detecting poly(ADP-ribose) in vitro and in vivo
Source: eLife. 2022 Apr 27;11:e72464. doi: 10.7554/eLife.72464 (PMC9045816; doi:10.7554/eLife.72464)
Supplement: Transparent reporting form [file elife-72464-transrepform1.docx]

***eLife’s* transparent reporting form**

We encourage authors to provide detailed information *within their submission* to facilitate the interpretation and replication of experiments. Authors can upload supporting documentation to indicate the use of appropriate reporting guidelines for health-related research (see [EQUATOR Network](http://www.equator-network.org/%20)), life science research (see the [BioSharing Information Resource](https://biosharing.org/)), or the [ARRIVE guidelines](http://www.plosbiology.org/article/info:doi/10.1371/journal.pbio.1000412) for reporting work involving animal research. Where applicable, authors should refer to any relevant reporting standards documents in this form.

If you have any questions, please consult our Journal Policies and/or contact us: [editorial@elifesciences.org](mailto:editorial@elifesciences.org).

**Sample-size estimation**

- You should state whether an appropriate sample size was computed when the study was being designed
- You should state the statistical method of sample size computation and any required assumptions
- If no explicit power analysis was used, you should describe how you decided what sample (replicate) size (number) to use

Please outline where this information can be found within the submission (e.g., sections or figure legends), or explain why this information doesn’t apply to your submission:

We did not use power analyses to determine the replicate numbers. The rationale for deciding on the number of replicates can be found in the next section (“Replicates”). The information on replicates can be found in the Methods and Figure Legends, as indicated in the next section.

**Replicates**

- You should report how often each experiment was performed
- You should include a definition of biological versus technical replication
- The data obtained should be provided and sufficient information should be provided to indicate the number of independent biological and/or technical replicates
- If you encountered any outliers, you should describe how these were handled
- Criteria for exclusion/inclusion of data should be clearly stated
- High-throughput sequence data should be uploaded before submission, with a private link for reviewers provided (these are available from both GEO and ArrayExpress)

Please outline where this information can be found within the submission (e.g., sections or figure legends), or explain why this information doesn’t apply to your submission:

We reported the details of replicates in Figure Legends.

The mice that became moribund at the time of measurements had to be removed from the xenograft studies in adherence with IACUC guidelines.

We used the ROUT analysis using Graphpad Prism to identify and remove the outliers in the immunofluorescence described in Figure 5D. We included this in the methods.

We do not have any high throughput sequencing data that require prior submission.

**Statistical reporting**

- Statistical analysis methods should be described and justified
- Raw data should be presented in figures whenever informative to do so (typically when N per group is less than 10)
- For each experiment, you should identify the statistical tests used, exact values of N, definitions of center, methods of multiple test correction, and dispersion and precision measures (e.g., mean, median, SD, SEM, confidence intervals; and, for the major substantive results, a measure of effect size (e.g., Pearson's r, Cohen's d)
- Report exact p-values wherever possible alongside the summary statistics and 95% confidence intervals. These should be reported for all key questions and not only when the p-value is less than 0.05.

Please outline where this information can be found within the submission (e.g., sections or figure legends), or explain why this information doesn’t apply to your submission:

(For large datasets, or papers with a very large number of statistical tests, you may upload a single table file with tests, Ns, etc., with reference to sections in the manuscript.)

The number of samples is indicated in the Methods and the Figure Legends.

For cell-based endpoint assays, we used at least n=3 biological replicates defined as temporally independent platings of cells from different cell passages. We chose this number since it is customary in the literature for these types of assays. The number of samples is indicated in the Methods and/or the Figure Legends.

**Group allocation**

- Indicate how samples were allocated into experimental groups (in the case of clinical studies, please specify allocation to treatment method); if randomization was used, please also state if restricted randomization was applied
- Indicate if masking was used during group allocation, data collection and/or data analysis

Please outline where this information can be found within the submission (e.g., sections or figure legends), or explain why this information doesn’t apply to your submission:

The mice used for xenograft experiments were assigned randomly into groups of treatments: Vehicle, Niraparib, and PARG inhibitor. This information is provided in Methods.

**Additional data files (“source data”)**

- We encourage you to upload relevant additional data files, such as numerical data that are represented as a graph in a figure, or as a summary table
- Where provided, these should be in the most useful format, and they can be uploaded as “Source data” files linked to a main figure or table
- Include model definition files including the full list of parameters used
- Include code used for data analysis (e.g., R, MatLab)
- Avoid stating that data files are “available upon request”

Please indicate the figures or tables for which source data files have been provided:

**Figure 1 – source data.** Source data and uncropped images for western blots in Figure 1D, 1F and Figure 1- figure supplement 1C, 1I, and 1F.

**Figure 1 – figure supplement 1 – source data.** Excel file with source data for the graphs in Figure 1 and Figure 1 – figure supplement 1.

**Figure 2 – source data.** Excel file with source data for the graphs in Figure 2.

**Figure 2 – figure supplement 1 – source data.** Excel file with source data for the graphs in Figure 2 – figure supplement 1.

**Figure 3 – source data.** Excel file with source data for the graphs in Figure 3

**Figure 4 – source data.** Source data and uncropped images for western blots in Figure 4B, 4E. Excel file with source data for the graphs in Figure 4.

**Figure 4 – figure supplement 1 – source data.** Source data and uncropped images for western blots in Figure 4- figure supplement 1B. Excel file with source data for the graphs in Figure 4 – figure supplement 1.

**Figure 4 – figure supplement 2 – source data.** Excel file with source data for the graphs in Figure 4 – figure supplement 2.

**Figure 5 – source data.** Source data and uncropped images for western blots in Figure 5A. Excel file with source data for the graphs in Figure 5.

**Figure 6 – source data.** Excel file with source data for the graphs in Figure 5

**Figure 7 – source data.** Excel file with source data for the graphs in Figure 6

**Figure 8 – source data.** Excel file with source data for the graph in Figure 7.

**Figure 8 – figure supplement 1 – source data.** Excel file with source data for the graphs in Figure 8 – figure supplement 1.
